# Quality of life, socioeconomic and psychological concerns in parents of children with tuberous sclerosis complex, STXBP1 and SYNGAP1 encephalopathies: a mixed method study

**DOI:** 10.3389/fped.2023.1285377

**Published:** 2023-11-09

**Authors:** María Salcedo-Perez-Juana, Domingo Palacios-Ceña, Ana San-Martín-Gómez, Ángel Aledo-Serrano, Lidiane Lima Florencio

**Affiliations:** ^1^ Research Group of Humanities and Qualitative Research in Health Science (Hum&QRinHS), Department of Physical Therapy, Occupational Therapy, Physical Medicine and Rehabilitation, Universidad Rey Juan Carlos, Madrid, Spain; ^2^ Epilepsy and Neurogenetics Programme, Vithas Madrid La Milagrosa University Hospital, Vithas Hospital Group, Madrid, Spain; ^3^ Research Group of Manual Therapy, Dry Needling and Therapeutic Exercise (GITM-URJC), Department of Physical Therapy, Occupational Therapy, Physical Medicine and Rehabilitation, Universidad Rey Juan Carlos, Madrid, Spain

**Keywords:** neurodevelopmental disorders, genetic epilepsy, pediatrics, carers, tuberous sclerosis complex, STXBP1, SYNGAP1, mixed methods research

## Abstract

**Background:**

Developmental and Epileptic Encephalopathies (DEEs) occur in childhood and are associated with severe epileptic seizures and neurological impairment. The aim of this study was to combine quantitative and qualitative methodologies to comprehensively describe factors related to quality of life, impact on the family and psychosocial factors in parents of children with TSC, STXBP1 and SYNGAP1 variants.

**Methods:**

A convergent parallel mixed design including parents of children with DEE. In the cross-sectional study, 20 parents (10STXBP1, five SYNGAP1, five TSC) were given questionnaires on quality of life, impact on the family and psychological factors. In the descriptive qualitative study, in-depth interviews were conducted with 18 parents (nine STXBP1, five TSC, four SYNGAP1) using a semi-structured questionnaire. A thematic analysis was carried out. The results of the two studies were combined by showing similarities and differences through tables, figures, accounts, and joint displays.

**Results:**

In terms of quality of life, the integrated results were consistent in highlighting the importance of family interaction, although in the qualitative section the influence of the relationship between the children's siblings, the relationship with health professionals and the difficulties in obtaining public aid were highlighted. In terms of impact, the integrated results show that the illness has a significant impact on the family; the financial burden is highlighted, and the experience of the illness is discussed in depth. Finally, the psychological aspects, symptoms such as anxiety, stress and strain, were consistent. Most of the participants reported sleep disturbance, as identified in the questionnaire, although not mentioned in the interviews.

**Conclusions:**

The combined results of the mixed method provide an in-depth analysis of the impact of DEEs on parents of children with STXBP1, SYNGAP1 and TSC.

## 1. Introduction

Developmental and Epileptic Encephalopathies (DEEs) are a group of rare diseases that present in childhood, with severe and difficult-to-treat epileptic seizures associated with learning difficulties, behavioral problems, and motor impairment (1–3). These diseases are also associated with high mortality and morbidity (3). In recent years, genetic variants in epilepsy have been identified, such as STXBP1, SYNGAP1, tuberous sclerosis complex 1 and 2 (TSC), SCN1A, KCNQ2, CDKL5, GNAO1, PCDH19, SHANK3 and Dup 15, which cause genetic epilepsy with onset in the first three years of life (4).

Severe epileptic seizures and neurodevelopmental problems (cognitive, motor) are common in DEEs, however, symptoms and their response to treatment vary (1, 2). For example, the STXBP1 variant has a very early onset, with refractory seizures, movement disorders and severe learning disabilities (4). TSC is characterized by early-onset epilepsy with associated neurodevelopmental problems, and the SYNGAP1 variant presents with refractory myoclonic seizures, absences, and varying degrees of autism spectrum disorder (4, 5). This variability in symptoms and their management has a major impact on the parents and families of children with DEEs (6, 7). Children affected by DEEs and their families experience difficulties with diagnosis and genetic identification, carer burden, and financial and social difficulties related to the disability (6, 7). These difficulties in caring for children with DEEs cause significant physical (exhaustion), mental (stress, anxiety, insomnia) and social (lack of resources) strain on parents, which can lead to health problems and reduced quality of life (6, 8–12). The European Commission, through the European Joint Programme on Rare Diseases (13), and the Orphanet network initiative (14) for rare diseases, has highlighted the need for further research into the diagnosis, treatment, and impact of rare diseases on patients, their families, and their social environment.

Therefore, the aims of this mixed methods research were to: describe the quality of life, family impact and psychosocial factors of parents of children with TSC, STXBP1 and SYNGAP1 variants; describe their experiences on these dimensions; and combine the findings to gain a better and broader understanding of the impact of the illness from the parents' perspective. To the best of our knowledge, this is the first study to attempt to describe the impact of DEE on parents of children with TSC, STXBP1 and SYNGAP1 using mixed methods research to analyze the impact of DEE from the parents' perspective.

## 2. Material and methods

### 2.1. Design

A convergent (concurrent) parallel mixed methods approach was used (15–17) (Table 1, Supplementary File S1). This included a quantitative (QUAN) observational cross-sectional study and a qualitative (QUAL) descriptive study, with data collection and analysis of both methods conducted simultaneously. The aim of a mixed methods research is to provide in-depth and contextualized answers to health science questions, by combining different approaches to overcome the limitations of a single method or methodological perspective (18). Previous studies (19–22) have demonstrated the use of mixed methods research to analyze the experiences of parents of children with rare neurodegenerative diseases such as MPS IIIA and Dravet (11, 20), to improve the health care and assessment of children with TSC (21, 23), and to assess the management and support of families with children with spinal muscular atrophy (19, 22).

**Table 1:** Convergent (concurrent) parallel mixed method study summary.

Study	Component	Sampling	Participants	Data collection	Analysis
Convergent mixed study	An observational cross-sectional study	Non-probability, purposive sampling, with consecutive inclusion of all cases that met the inclusion criteria.	Parents (mothers and fathers) of children genetically diagnosed with DEEs, TSC, STXBP1, SYNGAP1 variants	Sociodemographic and clinical variables. Quality of life: *Short-Form Health Survey, Beach Center Family Quality of Life Scale* Parents’ perceived impact: *The Impact on Family Scale, The Impact of Paediatric Epilepsy Scale* Psychological factors: *Beck Depression Inventory, State-Trait Anxiety Inventory, Pittsburgh Sleep Quality Index*	Descriptive analysis
	A descriptive qualitative study	Purposive sampling (maximum variation sampling technique)		In-depth interview based on guide questions	An inductive thematic analysis of participantś narratives.

Five researchers (three women) participated in this study, including one researcher nurse (DPC), three physiotherapists (LLF, MSPJ, ASMG), and one neurologist (AASN). None were involved in clinical activity, nor did they have any prior relationship with the patients included. Two researchers (ASMG, AAS) had clinical experience with DEEs. The National Institutes of Health guidelines for mixed methods research in health sciences were followed (24). In addition, the observational study followed the STROBE recommendations (25), and the qualitative study followed the SRQR and COREQ recommendations (26, 27).

### 2.2. Observational cross-sectional study (QUAN)

Cross-sectional studies are observational in nature and provide a snapshot of the characteristics of the study subjects at a single point in time. However, they do not have a follow-up period and cannot provide a cause-effect relationship (25).

#### 2.2.1. Sample and eligibility criteria

Parents of children with the TSC, STXBP1 and SYNGAP1 variants were recruited from the respective associations in Spain. Participants were recruited and assessed between February 2023 and July 2023.

As DEEs are rare diseases, the number of cases of each of the variants in Spain is low and/or may be underdiagnosed (28). In Spain, the most recent estimation of prevalence is 1906 for TSC (29), 20 for STBX1 (30) and 38 for SYNGAP1 (31). Non-probability convenience sampling was used, with consecutive inclusion of all available individuals who met the inclusion criteria.

Inclusion criteria: (a) Parents (mothers and fathers) of children diagnosed (with genetic diagnosis) with TSC, STXBP1, SYNGAP1 variants; (b) Parents of children with DEEs (afore mentioned variants) aged between 4 and 10 years (both included); (c) Parents living in Spain and belonging to one of the following patient associations: *Asociación española de esclerosis tuberosa, Asociación del síndrome STXBP1 and Asociación SYNGAP1 España*.

Exclusion criteria: (a) Parents who did not wish to participate in the study; (b) Parents of children with DEEs with different genetic variants; (c) Parents of an affected child who was undergoing an acute medical process that required hospital admission during the study; d) Parents of a child who did not present epileptic seizures in the previous month.

#### 2.2.2. Sociodemographic and clinical variables

The sociodemographic variables were age and sex of the parent, parent's educational level, number of children, age, and sex of the son/daughter, affected gene, age of onset of epileptic seizures, time to diagnosis. The following clinical variables were recorded: active epilepsy (seizures in the last 3 months), hospital admission (last year), number of visits to the hospital emergency department (last year), conduct disorder, and number of current anti-epileptic drugs.

#### 2.2.3. Quality of life

The Short-Form Health Survey (SF-12-v2) was used to assess parents' perceived quality of life (32, 33). This scale has 12 items assessing physical and mental health over the past 4 weeks, with scores ranging from 0 (worst) to 100 (best). Scores < 50 for the mental and physical components were considered below the norm for the general Spanish population (32).

The Beach Center Family Quality of Life (BCFQOL) Scale (34, 35), one of the most widely used scales in children with disabilities (36), was used to assess the family's quality of life. It consists of 25 items with five subscales: Family Interaction; Parenting; Emotional Wellbeing; Physical/Material Wellbeing; and Disability. The mean of the total item scores is obtained for each factor, on a Likert scale from 1 (very dissatisfied) to 5 (very satisfied). Higher scores indicate a better quality of life for the family.

#### 2.2.4. Parental perceived impact

The impact of the illness on the family was assessed using the Impact on Family Scale questionnaire (37, 38), which consists of 24 items with a 4-point Likert scale response. Four subscales are included: family/social impact (items 1–9, 9–36 points); personal impact (items 10–15; 6–24 points); experience of the illness (items 16–20; 5–20 points) and financial burden (items 21–24: 4–16 points). The score for each subscale can be reported separately or as an overall impact score (24–96 points), with higher scores indicating lower impact.

The Impact of Paediatric Epilepsy Scale (IPES) (39, 40) was also used. This scale enables parents to rate the influence of epilepsy on the daily life and quality of life of the family and child, currently and over the past 3 months. It considers 11 areas: general health, relationships within and outside the family, number of activities, schoolwork, self-esteem, loss of hope and family activities. They are scored from “very much” (3 points) to “not at all” (0 points), with a range of 0 to 33 points. Higher scores indicate a greater impact.

#### 2.2.5. Psychological factors

The Beck's Depression Inventory (BDI-II) (41, 42) and the State-Trait Anxiety Inventory (STAI) (43, 44) were used. The BDI-II consists of 21 questions to identify depression and is scored from 0 to 3 points. The final score determines the degree of depression identified: minimal (0–13 points), mild (14–19 points), moderate (20–28 points) and severe (29–63 points) (41, 42). The STAI assesses symptoms of anxiety with 40 items, with response options ranging from “not at all” to “very much” (from 0 to 3 points) (43, 44). The STAI is divided into two subscales, STAI-state and STAI-trait, with higher scores indicating greater state or trait anxiety.

The Pittsburgh Sleep Quality Index (PSQI) was also used (45, 46). It assesses sleep quality over 1 month and includes 19 items that form 7 component scores: subjective sleep quality, sleep latency, sleep duration, habitual sleep efficiency, sleep disturbances, use of sleep medication, and daytime dysfunction. The sum of the scores for each component is converted into an overall score (0–21 points), with a higher score indicating poorer sleep quality. Scores of 5 points or more indicate poor sleep quality (45).

#### 2.2.6. Analysis

The descriptive analysis of the data was carried out (by LLF, ASMG). As we could not confirm the normal distribution of all data (using histogram, normal Q–Q plots, and Shapiro-Wilk test); the continuous variables were described using the median and interquartile range or the frequency of observations. The variables have been described considering a total sample, understanding that they are parents of children with DEE, in addition to the description of the variables by subgroup of each genetic variant (TSC, STXBP1, SYNGAP1). No inferential analysis was performed due to the small sample size of the subgroups.

### 2.3. A qualitative study (QUAL)

A qualitative descriptive study was conducted (47). This type of qualitative design provides a rich description of the phenomenon of interest under study, informed by the experiences of the participants. Also, a descriptive qualitative study can help identify an event or critical situation (47).

#### 2.3.1. Participants and sampling strategies

Inclusion/exclusion criteria were the same as for the observational study. In the present qualitative study, a purposive sampling approach was used (48) based on the maximum variation sampling technique (49). This technique is used when the researcher wants to (a) select a purposive sample that is as representative as possible of a broader group of cases or (b) make comparisons between different types of cases. In the present study, the criterion to ensure variation was the different genetic variants of DEEs. In qualitative research there are also a variety of proposals for justifying and determining sample size (50, 51). Furthermore, there is no formula for calculating the sample size in advance (47). Due to this variability of criteria and the unavailability of many cases of each variant, the authors established the sample size based on pragmatic considerations (difficulty accessing participants because it is a RD). As a result, all available cases of each variant were included to enrich the data.

#### 2.3.2. Data collection

In-depth semi-structured interviews were conducted (48) with an open-ended follow-up question to collect the detailed descriptions (52). Table 2 Semi-structured interview question guide.

**Table 2:** Semi structured interview guide questions.

Research areas	Questions
Illness	What is it like to live with a child who has DEE? What is most significant to you?
Diagnosis	What was the process like leading up to the diagnosis? What was the most significant aspect of this process?
Symptoms	When did your child's symptoms begin to appear? What are the most significant for you? What factors, or situations, affect the symptoms?
Treatment	What is most significant about the treatment for you? How does it affect or limit your child's daily life? What side effects impact most on your child's life? What are your hopes for your child's treatment? Do you use any coping strategies to deal with the symptoms of the illness?
Adherence to treatment	What obstacles and facilitators affect your ability to adhere to the treatment recommended by your doctor? Have you tried other treatments?
Family planning	How does the illness affect your life as a couple, and has your view on having children changed?
Family relationships	How does the illness affect your family life? Has it had any impact or repercussion on your relationship with other family members (grandparents, siblings, etc.)?
Resources and access to services	What obstacles and facilitators do you notice when trying to access resources for your child's care? Has your child's illness had any impact on your financial situation? What obstacles and facilitators have you experienced when trying to access health and social services?
Hopes for the future	What are your hopes for your child, for the illness and its progression?

The interview question guide was developed based on previous studies concerning DEE (11, 12, 53–56) where a pilot test was conducted (52, 57). All interviews were conducted by two researchers (MSPJ and DPC), with experience in developing qualitative studies using in-depth interviews and not involved in the care of the participants' children. All interviews (*n* = 18; 9 STXBP1, 5 TSC, 4 SYNGAP1) were audio-recorded, recording a total of 1,632 min of interviews (the average duration of each interview was 90.67 min).

#### 2.3.3. Data analysis

Inductive thematic analysis was used (47, 48, 58). Full transcriptions were made of each of the interviews. Thematic analysis consisted of identifying the most descriptive content to obtain meaningful units (codes), and then reducing and identifying the most common meaningful groups (categories) (47, 48, 58). Thus, groups of codes were formed, i.e., similar points or content that enabled the emergence of themes that offered a detailed perspective of the study participants. The analysis was carried out separately for each interview (by DPC, MSPJ). Joint team meetings were held to combine the results of the analysis. In the case of a divergence of opinion, the identification of the results was based on consensus among the members of the research team. For the analysis, Excel spreadsheets were used to organize and share the coding process. See Supplementary File S2 Data analysis and coding procedure.

#### 2.3.4. Rigor

The application procedures used to control the trustworthiness of the qualitative study are described in Supplementary File S3. Trustworthiness criteria (59).

### 2.4. Integration procedure for quantitative and qualitative content

Data were integrated through a mixed concurrent design, where simultaneous data collection and analysis of the QUAN and QUAL studies were carried out (60), and the findings were interpreted and reported through accounts and joint displays (61–63). Data integration was performed after the analysis of each study by the four investigators (LLF, ASMG, DPC, MSPJ). The results of The QUAN and QUAL are reported in the same section and a table has been used for joint display of the data integration.

## 3. Results

The results are reported in the following order: (1) quantitative and qualitative results, and (2) mixed method findings (integration) (61–63). The accounts or narratives for each dimension studied, which explain the qualitative results, can be found in Supplementary File S4. Joint display of quotes organized by study dimensions.

### 3.1. Sociodemographic data

Twenty parents completed the questionnaires, and 18 parents participated in the interviews. The socio-demographic characteristics of the participants and the clinical characteristics of the children with DEE are shown in Table 3.

**Table 3:** Sociodemographic data of the parents of children with developmental and epileptic encephalopathy variants STXBP1 and SYNGAP1 and tuberous sclerosis complex and clinical variables of their child.

		Total sample (*n* = 20)	STXBP1 (*n* = 10)	SYNGAP1 (*n* = 5)	Tuberous sclerosis complex (*n* = 5)
Participant's characteristics					
Age^a^		39.5 (11.5)	36.0 (11.0)	43.0 (8.0)	40.0 (6.0)
Sex	Female	17 (85%)	9 (90%)	4 (90%)	4 (80%)
	Male	3 (15%)	1 (10%)	1 (10%)	1 (20%)
Educational level	Secondary school	7 (35%)	3 (30%)	2 (40%)	2 (40%)
	Higher education	13 (65%)	7 (70%)	3 (60%)	3 (60%)
Reduction of working hours		11 (55%)	6 (60%)	1 (20%)	4 (80%)
Number of children^a^		2.0 (1.0)	2.0 (0)	2.0 (1.0)	2.0 (1.0)
Place of residence	Urban	7 (35%)	2 (20%)	1 (20%)	4 (80%)
	Village	7 (35%)	5 (50%)	2 (40%)	0
	Not reported	6 (30%)	3 (30%)	2 (40%)	1 (20%)
Family Income (minimum wage^b^)	<1	1 (5%)	1 (10%)	0	0
	1–2	6 (30%)	4 (40%)	1 (20%)	1 (20%)
	3–4	9 (45%)	3 (30%)	3 (60%)	3 (60%)
	5–6	2 (10%)	1 (10%)	1 (20%)	0
	≥7	2 (10%)	1 (10%)	0	1 (2%)
Receiving Social aids^c^		18 (90%)	9 (90%)	4 (80%)	5 (100%)
Living with other members of family		0	0	0	0
Characteristics of children with DEE					
Age^a^		6.5 (3.5)	6.5 (4.0)	8.0 (1.0)	4.0 (1.0)
Sex	Female	10 (50%)	3 (30%)	3 (60%)	4 (80%)
	Male	10 (50%)	7 (70%)	2 (40%)	1 (20%)
Age at onset of seizures (months)^a^		4.5 (26.9)	0.3 (3.9)	39.0 (42.0)	5.0 (4.9)
Time to diagnosis (months)^a^		11.0 (15.0)	18.0 (16.0)	12.0 (14.0)	4.0 (1.0)
Active epilepsy	Yes	10 (50%)	4 (40%)	1 (20%)	5 (100%)
	No	10 (50%)	6 (60%)	4 (80%)	0
Hospital admission in the last year (*n*)	Yes	9 (45%)	6 (60%)	2 (40%)	1 (20%)
	No	11 (55%)	4 (40%)	3 (60%)	4 (80%)
Emergencies in the last year (*n*)^a^		1.0 (3.5)	1.0 (5.0)	1.0 (1.0)	1.0 (4.0)
Behavioral disorder	Yes	14 (70%)	5 (50%)	4 (80%)	5 (100%)
	No	6 (30%)	5 (50%)	1 (20%)	
Antiepileptic drugs (*n*)^a^		1.0 (2.0)	1.0 (2.0)	1.0 (1.0)	3.0 (1.0)
Drugs for other aspects (*n*)^a^		2.0 (2.5)	1,5 (1,0)	2.0 (3.0)	4.0 (1.0)

^a^ Median (interquartile range).

^b^ Minimum wage of 1,080€ at the time.

^c^ Social benefits included benefits for dependency and disability, housing costs, food, treatment and home adaptations.

### 3.2. Quality of life

#### 3.2.1. Quantitative data

**Figure 1:**
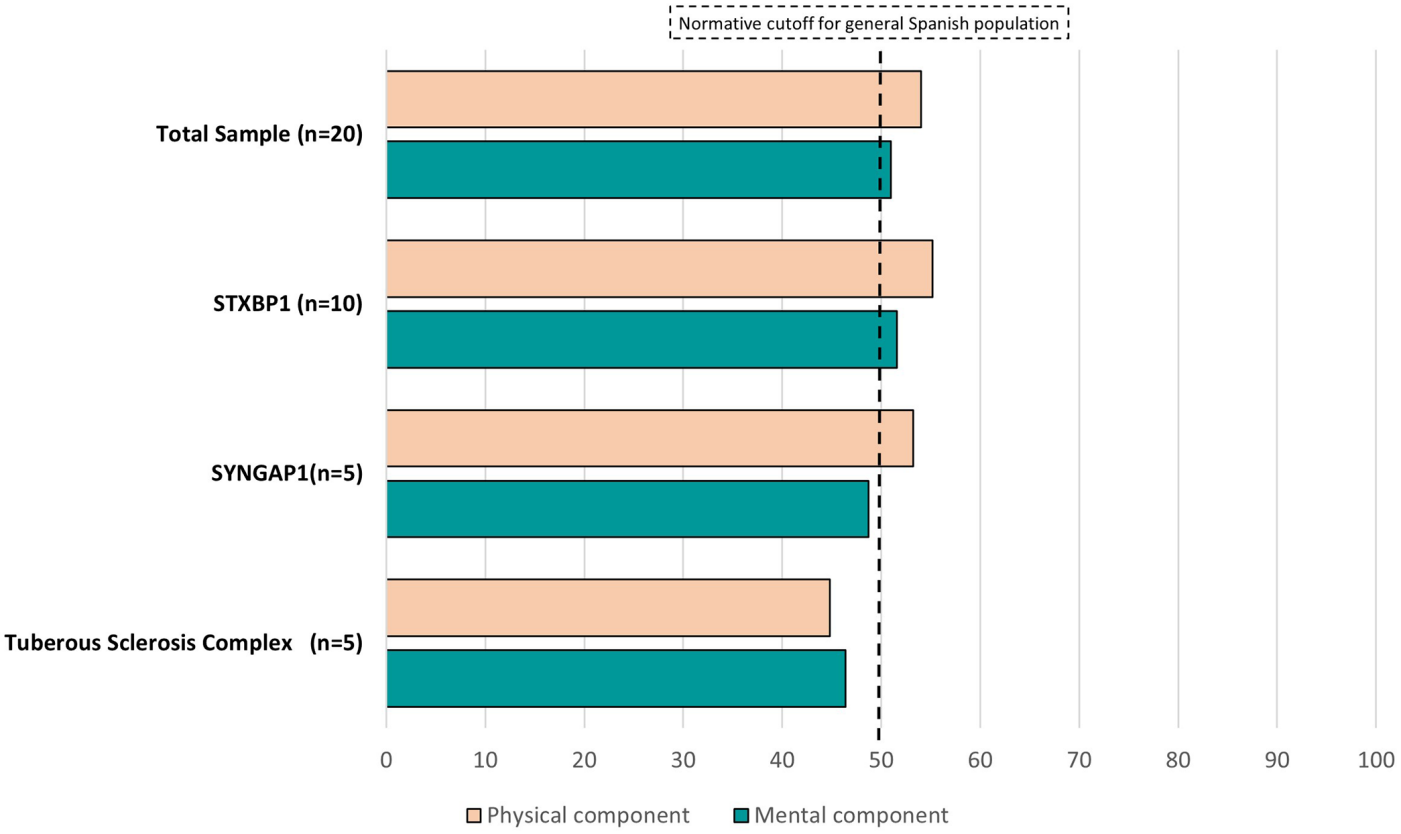
Quality of life, measured by the short-form health survey (SF-12), of the parents of children with developmental and epileptic encephalopathy variants STXBP1 and SYNGAP1 and tuberous sclerosis complex.

The median of SF-12 scores for the whole sample is close to the cut-off point of the norm value of 50 for the general Spanish population in both components, however, the mental component of group of parents of children with SYNGAP1 and the physical and mental components for the TSC group are below this cut-off (Figure 1 and Supplementary File S5).

**Figure 2:**
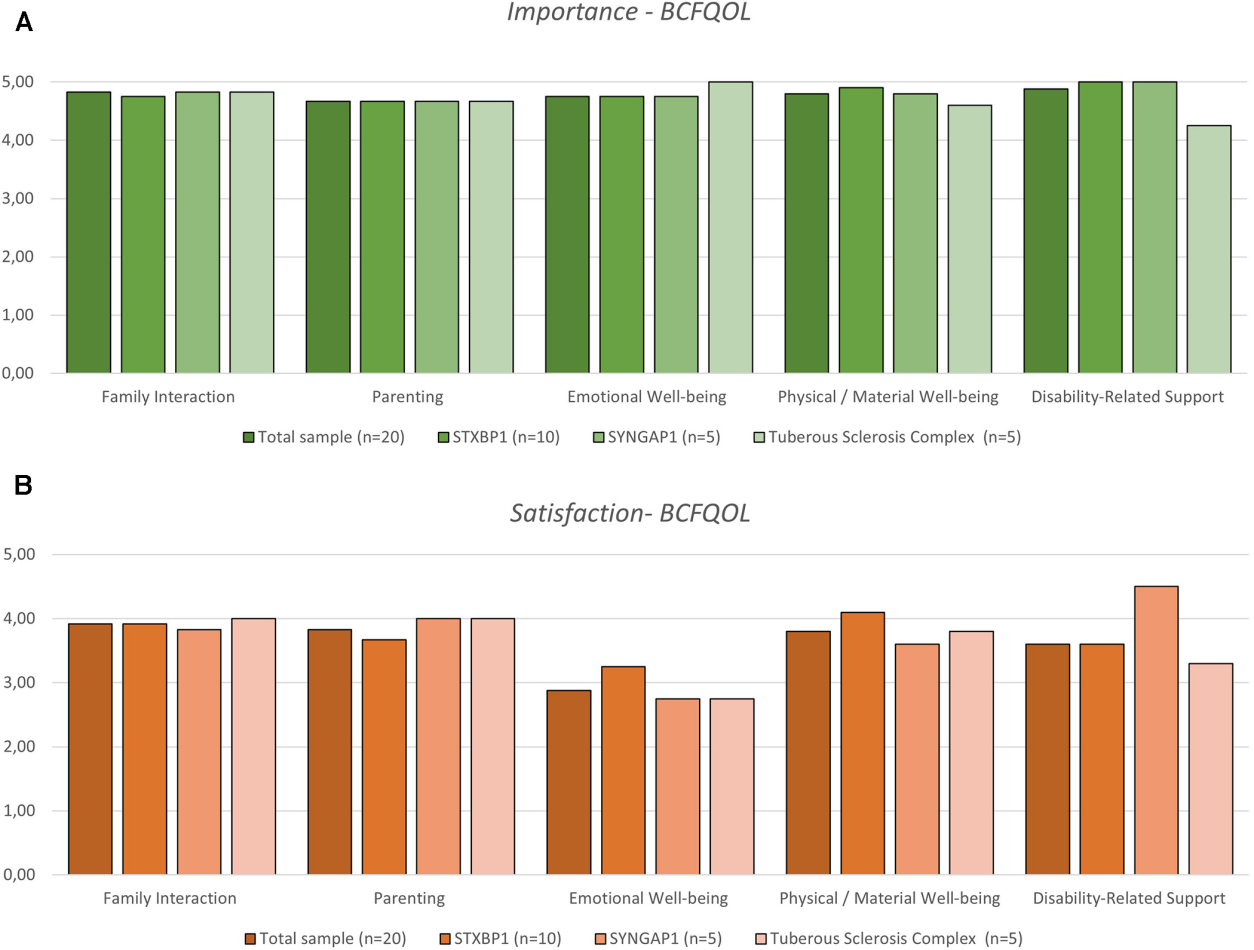
Family quality of life reported by the parents of children with developmental and epileptic encephalopathy variants STXBP1 and SYNGAP1 and tuberous sclerosis complex, measured by the beach center family quality of life (BCFQOL) scale. The parents’ perspective is divided by importance (**A**) and satisfaction (**B**) with each dimension of the scale.

In terms of family quality of life, according to the BCFQOL, family interaction and disability-related support are the dimensions that participants rated as the most important, although there was an even distribution of importance across all dimensions (Figure 2A, Supplementary File S5). Also, participants were most satisfied with the family interaction and least satisfied with the emotional wellbeing dimensions (Figure 2B, Supplementary File S5).

#### 3.2.2. Qualitative data

Qualitative interviews demonstrated the following contents regarding quality of life. Parents related that it was key to establish good relations within the family structure (parents-children) and to avoid the family rejecting their child. In all variants, there were cases of rejection (avoiding contact) or lack of understanding by the family (not recognizing the illness). For the parents, the remaining family (parents, siblings, grandparents, etc.) also had to experience their own grief, and take their own time to accept it. Conversely, there were also cases where the family was fully supportive and contributed towards the child's care. Moreover, it brings joy and relief to the parents when siblings accept the child with DEE; when they show love, support, and help; when there is a normal relationship; and when they know that they can count on the siblings in their absence.

All the parents rated the associations positively as a source of help and support, understanding, guidance and advice throughout the process.

There are positive experiences with professionals in all variants, however, most parents reported that the care should improve in terms of sensitivity and empathy of professionals, support for families, knowledge of the illness, concern for the children, and their difficulties. In children with SYNGAP1, parents considered that the process of obtaining a diagnosis should improve, together with the assessment of the information provided by parents about symptoms. This was especially the case when parents showed video recordings of epileptic seizures to professionals as evidence to support their claims. In TSC, parents felt that communication between professionals was lacking, also, they complained of insufficient follow-up of their children, the important role of the doctor as a guide for care, and the need to increase the time available for consultations, recommending that professionals should interact with children with DEE, and pay attention to them, not treat them like objects.

The obstacles identified by the parents included: restrictions on social assistance due to the lack of recognition of DEE as eligibility criterion, the high degree of bureaucracy, lack of information about resources, restricted access to buildings, waiting lists for public health care, and delays in care between specialists. In SYNGAP1, parents hired private professionals (physiotherapists, psychologists) and pointed out that children with learning and/or sensory disabilities had fewer opportunities. Some TSC cases highlighted the lack of coordination between public and private hospitals, which forced them to travel long distances to seek hospital help.

### 3.3. Impact on the family

#### 3.3.1. Quantitative data

**Figure 3:**
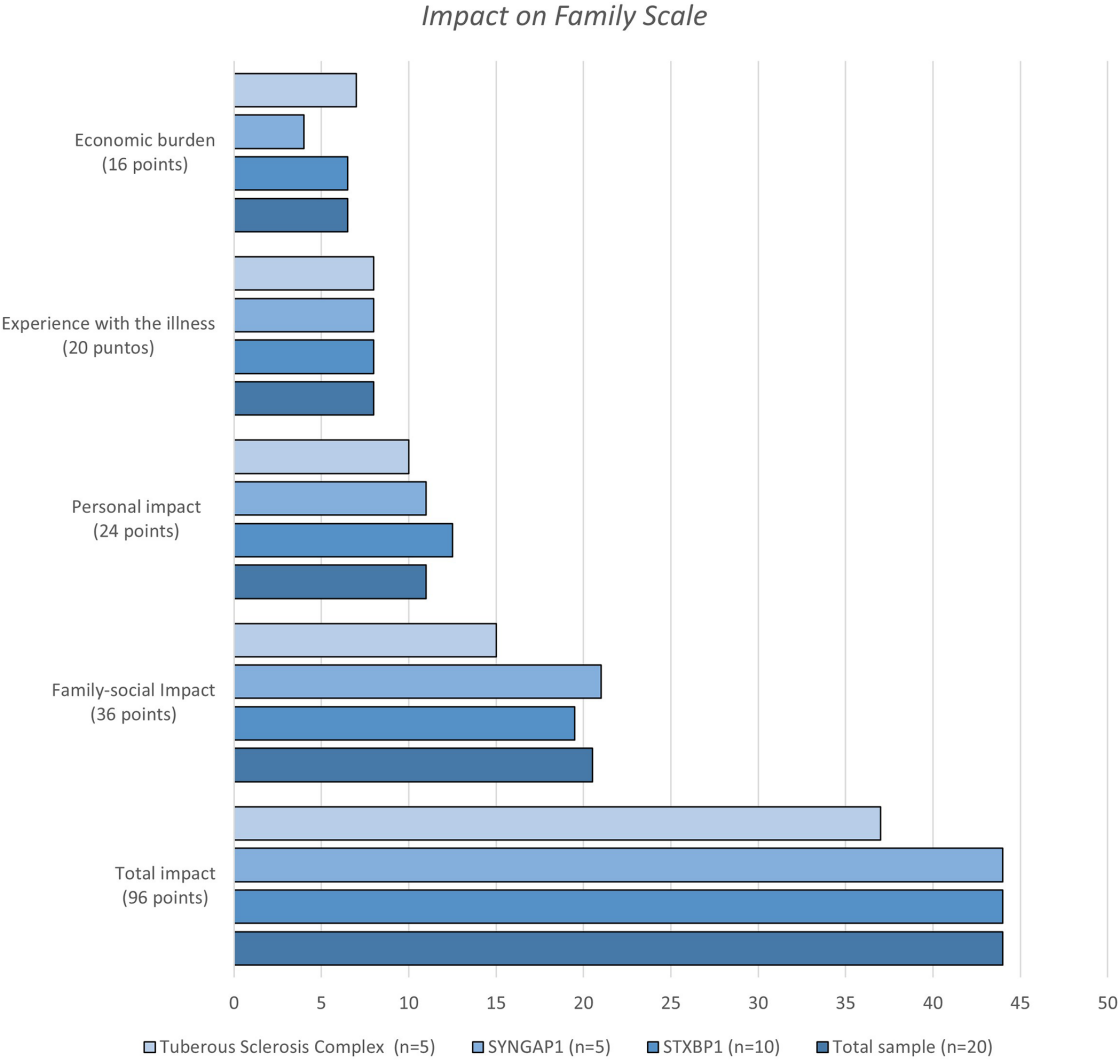
Impact of family scale scores of the parents of children with developmental and epileptic encephalopathy variants STXBP1 and SYNGAP1 and tuberous sclerosis complex.

The median scores of the Impact on Family Scale are shown in Figure 3 and in Supplementary File S5. Proportionally, the subscales “experience with the illness” and “economic burden” were the most affected in the total sample. In the total score, the subgroup of parents of children with TSC had the highest impact.

**Figure 4:**
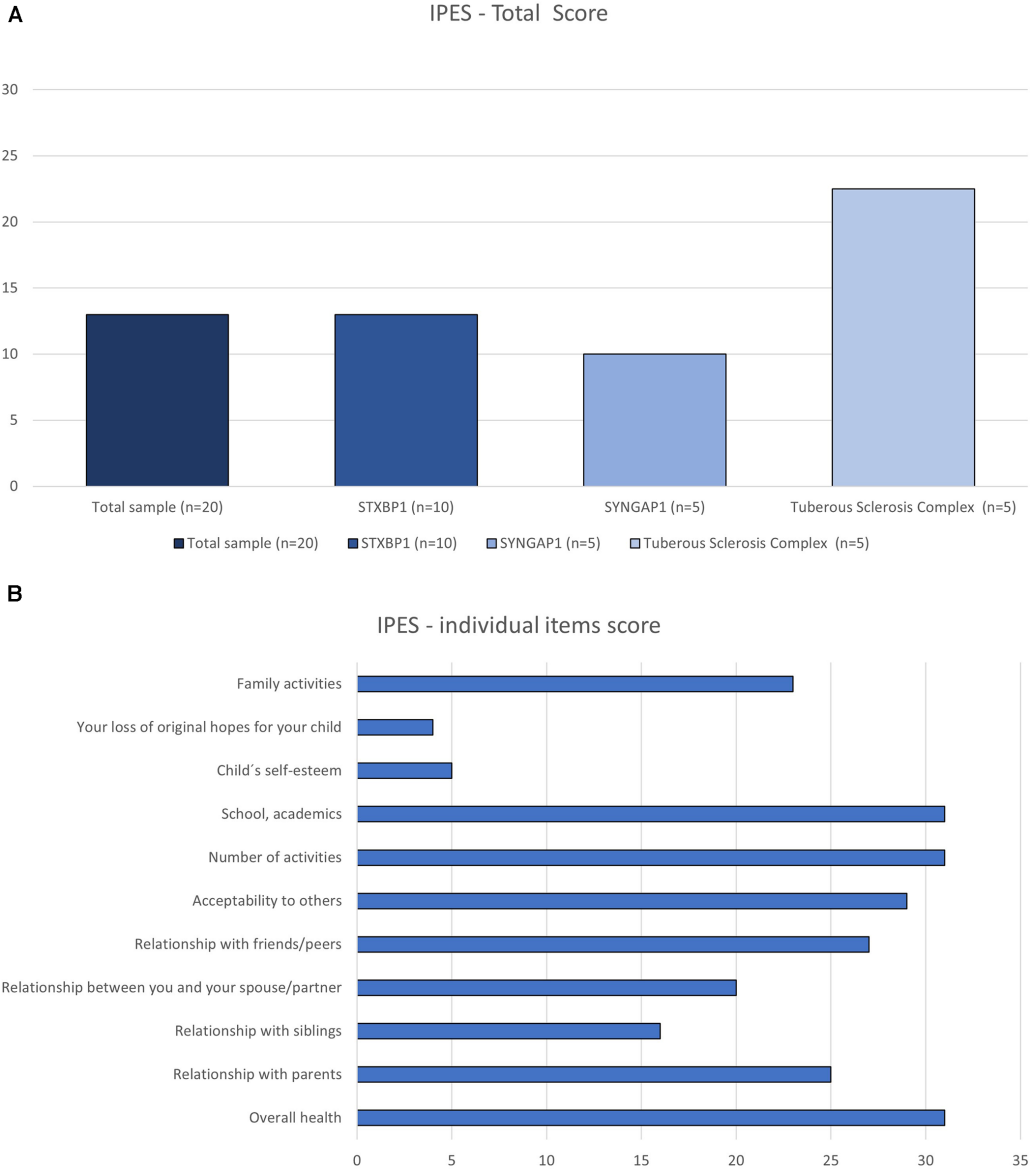
Impact of paediatric epilepsy scale (IPES) rated by the parents of children with developmental and epileptic encephalopathy variants STXBP1 and SYNGAP1 and tuberous sclerosis complex. Data is displayed as total score (**A**) and the sum of parents’ scores of each item on the scale (**B**).

Two participants answered “not applicable” to all 11 items of the Impact of Paediatric Epilepsy Scale and were not included in the descriptive analysis. The subgroup with the highest total score were the parents of children with TSC (Figure 4A, Supplementary File S5). For the total sample, the impact was higher in general health, number of activities and at school (Figure 4B). It is noteworthy that the low scores for self-esteem and loss of hope in the child are a result of the high rate of “not applicable” responses between 50%–55% of the sample for these two items.

#### 3.3.2. Qualitative data

The main change brought about by the illness was that all the parents' time and attention was focused on the children. All family life (work, leisure, school) revolved around them. All children required constant monitoring for symptoms, however, there were some specific features; in STXBP1, parents highlighted the difficulty of managing the child physically, whereas in SYNGAP1 and TSC, parents highlighted the difficulty of managing behavior, with harsh reactions (shouting), violent (hitting, pinching), and angry behavior.

In all variants, there were changes in social relationships and friendships. In some cases, a lack of understanding of the illness led to distancing, while in others, the new family situation meant that they no longer attended social and/or fun gatherings.

In all variants, DEE also affected siblings. The main change was a reduction in time and activities shared with the parents. Nonetheless, there were also other consequences, such as psychological care for the siblings due to poor grief management, rejection, shame in being with the family, denial of responsibility for the care of the sibling with DEE in the future. However, other parents also found siblings to be supportive and helpful, mature, the most faithful and loving companions of the child with DEE. To lessen the responsibility on siblings, some parents (STXBP1) saved money to ensure the future care of their child with DEE and avoid becoming a financial burden on their siblings. Others reported the importance of ensuring that the child's siblings had their own space and life trajectory (TSC).

The parents of children with STXBP1 and SYNGAP1 variants reported difficulties in finding temporary carers to allow parents to perform other tasks and/or take a break. The reasons for this were: difficulty in managing children with DEE (“conduct disorder”), constant availability, lack of knowledge about the illness, and lack of training in managing epileptic seizures. In addition, some parents admitted that they found it difficult to leave their children in the care of others.

The parents talked about how they had learned to respect others, to appreciate the little things, to enjoy every moment and to be flexible in the face of unforeseen events.

There was conflict between the couple due to reduced time together, physical and mental exhaustion, and overload of care responsibilities. This led to distancing, stress, tension, and arguments. The participants reported how this phase could be overcome, resulting in a strengthening of the couple and a greater bond.

Regardless of the variant, some participants stated that they would consider having another child, while others clearly refused having another child. The reasons for having another child included: wanting to be a mother again, wanting a child without illness, wanting the child with DEE to have a carer in the future, and wanting a new lease of life. Conversely, reasons for not wanting to have another child were because they did not want to spend less time with their current children, because they did not have the time, because of a lack of money, and because they were afraid another child would have DEE again.

Financial burden appeared in all variants. Families paid for additional treatments (physiotherapy, occupational therapy), medicines, special diets (ketogenic diet), orthopedic products, home adaptations to cope and private consultations with medical specialists. In all variants, one of the parents had to ask for a reduction in working hours, in order to have time to care for their child, adding to the financial burden.

### 3.4. Psychological factors

#### 3.4.1. Quantitative data

Table 4 displays the descriptive analysis of anxiety, depression, and sleep quality. Up to 40% of the participants presented at least mild signs of depression. Moreover, 75% had poor sleep quality. Parents of children with TSC presented a higher median score for the STAI-trait, BDI-II and the PSQI; whereas SYNGAP1 and STXBP1 subgroups presented more state-anxiety.

**Table 4:** Psychological aspects (anxiety, depression and sleep quality) of the parents of children with developmental and epileptic encephalopathy variants STXBP1 and SYNGAP1 and tuberous sclerosis complex.

		Total sample (*n* = 20)	STXBP1 (*n* = 10)	SYNGAP1 (*n* = 5)	Tuberous sclerosis complex (*n* = 5)
State-Trait Anxiety Inventory (STAI)—State^a^		25.5 (11.0)	26.5 (11.0)	26.0 (7.0)	23.0 (11.0)
State-Trait Anxiety Inventory (STAI)—Trait^a^		22.5 (5.0)	21.0 (5.0)	23.0 (3.0)	26.0 (4.0)
Beck Depression Inventory (BDI-II)	Score^a^	11.5 (12.0)	9.5 (16.0)	12.0 (8.0)	20.0 (8.0)
	Minimal	12 (60%)	7 (70%)	3 (60%)	2 (40%)
	Mild	1 (5%)	0	1 (20%)	0
	Moderate	6 (30%)	3 (30%)	0	3 (60%)
	Severe	1 (5%)	0	1 (20%)	0
Pittsburgh Sleep Quality Index (PSQI)	Score^a^	8.5 (5.5)	7.0 (4.0)	9.0 (3.0)	11.0 (7.0)
	Poor sleep quality (*n*/%)	15 (75%)	7 (70%)	4 (80%)	4 (80%)

^a^ Data are presented by median and interquartile range.

#### 3.4.2. Qualitative data

Many parents reported feeling anxious about their children's future as they grew older. In the SYNGAP1 subgroup, they worried that their child would become unmanageable, and that institutionalization would be considered. Parents acknowledged feelings of loneliness when they felt overwhelmed and overburdened by tasks (care, work, managing consultations, etc.). Parents experienced a constant feeling of uncertainty as the child's health can change suddenly. They live with stress because they cannot plan anything, and therefore they feel uncertain about the treatment, and are frustrated by their child's dependency. In some cases of TSC, parents described feeling tied down by the illness, even feeling like they were imprisoned. Many were continually exhausted by the constant vigilance their children required.

In all variants, there was no hope for the future, no goals, no long-term planning, living from day to day, focusing on daily achievements. They also described a period of prolonged mourning and/or grief, because of the impact of the illness and all the lost hopes and dreams. Parents in all variants described their experience as hard, painful, and intense, however, they acknowledged that certain rewarding moments of great happiness exist, related to the great love they feel towards their sick children, family togetherness and learning to cope with the illness together.

Parents of children with all variants experienced some sense of guilt. For example, when they must travel (for work) they feel that they are abandoning their children, or because they feel responsible for making their siblings live with the illness (STXBP1). When the children are in hospital and the parents go home to rest, or when they think that they may have been responsible for the illness (SYNGAP1). They also felt guilty when siblings had problems at school, or for not spending enough time with them (TSC). In extreme moments of feeling emotionally exhausted and in crisis, some parents wished their child would die because it would be a liberation. In these cases, this thought was accompanied by a strong sense of guilt. The emotions are so intense that, during a crisis, not only do parents wish their child were dead, they also become disconnected from their care and/or reject them. One participant (C3) described how life was unbearable when their child with SYNGAP1 shouted and hit them when he was angry. Another parent (B1) reported that there were times when they rejected their child with TSC, and at these times they were unable or unwilling to care for their child.

In all variants, reducing working hours to care for their children was a difficult decision, and was accompanied by anger, feelings of worthlessness, loss of job opportunities, and difficulty in accepting leaving work (STXBP1). Parents also experienced feelings of shame because they felt that they did not deserve their salary, together with frustration, and fear of being fired (TSC). In contrast, there were cases in STXBP1 and SYNGAP1 where the reduction in hours was experienced positively, as they had more time for their child and partner.

### 3.5. Mixed method findings (integration)

The results of the integration showed similarities and differences (56, 58, 59) between QUAL and QUAN results (Table 5).

**Table 5:** Combined display of the quantitative and qualitative findings.

Outcomes	Quantitative findings	Parents' experiences
Quality of life	*SF-12:* • Quality of life scores close to norm values for the Spanish population. • The physical and mental effect of the illness in STXBP1 and TSC is similar, though in SYNGAP1 physical involvement is more prominent than mental involvement. *Importance BCFQOL:* • Family interaction and disability-related support are the most important aspects for parents. • For the three variants, the importance given to family interaction, parenting, emotional wellbeing, physical/material wellbeing and disability-related support is similar. *Satisfaction BCFQOL:* • Differences appear between the variants. SYNGAP1 shows less satisfaction in relation to the other two variants, in family interaction and physical/material well-being, and TSC shows less satisfaction in disability-related support.	• There are no accounts of physical condition limiting activities. Their activities are limited by organising their lives for childcare. The mental component is given greater consideration. • The importance of maintaining good relationships between family members and their family-social environment is highlighted. Special consideration is given to maintaining a good relationship with and feeling the support of the siblings of the child with DEE. • In all variants, the associations play an important role in providing support to families in dealing with the illness. • The presence of distancing or misunderstanding on the part of other family members (grandparents, cousins, etc.) is not a widespread behaviour. • One aspect of quality of life, which does not appear in the questionnaires used, is the relationship with health staff, and a large number of improvements in the care of parents and children with DEE are identified. • The perceived obstacles to being able to provide care for their child (public aid, access to health care, etc.) affect parents’ quality of life and force them to use their savings to cover their children's needs.
Impact on family	*Impact on Family Scale*: • The most impacted areas are “experience with the illness” and “economic burden”. Similar impact between variants on all subscales, but highest overall impact on the TSC variant. *Impact of Paediatric Epilepsy Scalee:* • TSC shows a greater impact than STXBP1 and SYNGAP1. • The most affected items included children's school and academic activity, number of activities, social life and acceptance, child's friends and peers, relationships with parents and general health.	• Intense and hard experience, which changes the whole personal and family life, also affecting the siblings of the child with DEE. The impact on siblings is variable, with both positive and negative effects that influence the relationship within the family. • The presence of distancing or misunderstanding on the part of friends or other people (at school, work, etc.) is not a widespread behaviour. • All personal and family activities revolve around the child with DEE. In STXBP1, the difficulty of handling the child physically is highlighted, and in SYNGAP1 and TSC disruptive behaviour is highlighted. • The couple is put to the test, numerous conflicts arise, which, once overcome, can strengthen the relationship. The desire to have another child can change. • There is a financial impact in all variants, which is exacerbated when one partner's working hours have to be reduced in order to care for the child.
Psychological factors	*STAI:* • SYNGAP1 is the variant with the most state- and trait-anxiety. *BDI-II:* • Forty percent of the sample have at least mild signs of depression. Higher scores for SYNGAP1 and TSC variants. *Pittsburgh Sleep Quality Index:* • Poor sleep quality in the majority of participants in all variants.	• In all variants parents describe the presence of anxiety, tension, stress, strain, and exhaustion affecting the couple and family relationship. • In all variants parents share concern for their child's health status, guilt, lack of future plans and no hope for a cure or improvement. • During periods of emotional crisis there are cases where parents may wish their child dead, disengage from their care and/or reject them. • In their accounts parents did not highlight sleep disturbances as a relevant element in their mental state.

## 4. Discussion

This mixed-method study provides a broader, in-depth perspective of parents with DEE. (1) In terms of quality of life, our findings suggest that individual questionnaires may not properly reflect their experience and family quality of life may be more appropriate; however, none of the quantitative tools addressed aspects such as the experience with professionals and the role of the associations, which are aspects that emerged from the interviews. (2) Results of QUAN and QUAL studies about impact on the family dimension were convergent, affecting familýs time and financial burden. QUAL also highlights the parent`s concerns regarding the siblings and the decision to have another child. (3) Psychological aspects, such as anxiety, depression, stress, guilty, anger and feelings of worthlessness were identified. Moreover, QUAN results demonstrate high rates of sleep disorders that were not mentioned by the parents during the interviews. This integrative perspective has never been addressed before in parents of children with the variants studied.

### 4.1. Quality of life

Previous studies on DEEs (12, 53) have shown how parents' lives “stop” to focus on their children, and day-to-day life is a constant adaptation requiring parents to be alert.

Carers of children with TSC have lower health-related quality of life (HRQL) than the general population (64–67), spending an average of 104 h per week caring. The more time spent caring, the lower the family functioning (*p* = 0.01) and the lower their' HRQL (*p* = 0.03) (68). In addition, neuropsychiatric comorbidities in children with TSC were also associated with lower family functioning (*p* = 0.02) and carer HRQL (*p* < 0.01). This decrease in carer and family HRQL occurs because the daily routines of the entire family change as they focus on the needs of the sick child (65, 66), such as monitoring seizures, assessing risk of injury, managing disruptive behavior, and changing work shifts for medical appointments, affecting their daily planning or their social activities (8, 65, 66). Thus, carers need time for themselves to maintain their quality of life.

Our results show how the illness causes changes in relationships with family (grandparents, cousins, etc.). In DEEs, family relationships can be affected, with feelings of isolation, lack of support/understanding, and regular family crises (69, 70). Family of children with TSC reported that isolation allowed them to explore alternatives and/or to build a protective “glass dome” around the child (66). In other DEEs (70, 71), parents stopped attending family gatherings to avoid inappropriate looks or questions and lost contact with friends as well as ceasing to go on social outings.

The relationship with health professionals is not an item that appears in the quality of life and family impact questionnaires. Discrepancies in the treatment families expect from professionals may be due to professionals focusing on different aspects of the child's care than those expected by carers (72). Sullivan et al. (73) demonstrated how professionals prioritize motor and developmental delay, movement disorders and tremor in the care of children with STXBP1 over other problems (considered essential by carers) such as behavioral problems and nutrition. Similarly, in TSC, Zöllner et al. (72) showed that there were discrepancies in the prioritization of the management of psychiatric and neurological symptoms between physicians and carers. Previous studies (12, 54, 74) in DEEs have shown the importance of the relationship with the healthcare professional for parents. For parents of children with SCN1A, KCNQ2, CDKL5, PCDH19, and GNAO1, the relationship was negative when professionals prioritized bureaucracy, ignored information provided by the family, communicated without empathy, judged the quality of home care, did not relate to their children, and did not understand the illness (12).

The major role of support associations has already been reported in DEEs and rare diseases (70, 71, 75, 76) as they help and support families, act as a filter of information, allow sharing of experiences, and improve quality of life of families. However, access to associations should take place progressively to avoid information overload and stress. Graffigna et al. (66) described how families of children with TSC used the association as a primary form of support, information, and engagement with other families as they learned to accept their child's disability.

Our qualitative results showed obstacles for obtaining social support, which is in line with a previous report of parents of children with rare diseases and DEEs (55, 77–80). Bureaucracy is an ongoing struggle and parents feel powerless and sometimes forgotten by support providers (55, 79).

### 4.2. Impact on the family

The multiple symptoms and the disability associated with DEEs impact parents' lives in multiple ways. A conceptual model of the STXBP1 shows that the symptoms that most affected parents were developmental delay and behavioral problems, however, their emotions are also affected and their daily activities are limited (73). For carers of children with TSC, the most bothersome symptoms are the seizures, disruptive behavior, and cognitive problems (72). For SYNGAP1, most parents were concerned about language impairment, behavioral problems, lack of autonomy in their children, and increasing family and financial burden (81). Also, in SYNGAP1, parents struggled with distress-related behaviors in everyday life, where children displayed frustration and aggression when they were denied something, did not get their own way or were unable to understand the situation. Very often this aggression could lead to violence towards themselves or towards others (5).

Despite this situation, parents of this study and carers of children with other DEEs are able to experience happiness and joy, as well as learn from their children (53, 70, 72, 80, 82). This change in their outlook on life seems to be related to a better knowledge of the illness, its management, and their expectations, a process of readjustment in which they manage to reach an acceptable level of emotional well-being.

Nonetheless, living with these DEEs may negatively impact the couple's relationship, leading to separation and/or divorce (66). Caring for children with DEEs involves making decisions, changing roles, and limiting activities that provoke conflict in the couple and affect their sex life (6, 70). The key to strengthening the couple is communication, “team building”, respect, patience and recognizing that both partners are suffering (70). One source of conflict was the decision to have more children. Doubt about having another child is also seen in Phelan-McDermid syndrome (PMS) (53, 54), where the decision was accompanied by fear and uncertainty.

The reduction of working hours observed in our participants for all variants is a common strategy described among carers of children with TSC (65, 72), carers' professional careers and/or productivity is affected because they must leave work (resignation or dismissal) and/or reduce their working hours in order to care for their sick children. Consequently, there is an increase in the economic burden, which is already large considering the costs of orthopedic products, nappies, and arranging treatments (physiotherapy, occupational therapy) (54, 80, 83).

In the case of TSC, Zöllner et al. (72) described how children with cognitive impairment and severe forms of epilepsy have a higher risk of hospitalization, admission to intensive care units and undergo more diagnostic procedures than the rest of the population, regardless of the health care system. In addition, these children require more effort and many expenses must be met to cover rehabilitation, physiotherapy, and speech therapy. In terms of direct costs, a patient with TSC incurs an average total cost of £12,681 (PPP-$17,629) over a three-year period, compared to £4,777 (PPP-$6641) per patient in the general population. Previous studies on Dravet (84, 85), reported a mean direct and indirect cost of €6,043 and €4,399 per year, respectively. The most significant financial burden was associated with hospitalization, care services, and anti-epileptic drugs. In addition, the cost increased if the child had frequent seizures, hospitalizations, home visits, severe symptoms and/or required nursing care (84, 85).

### 4.3. Psychological factors

The findings highlight parents' despair and lack of hope for the future. Previous studies (86–90) have shown that managing “hope” in parents of children with rare neurological disorders should be a priority for healthcare professionals. Feelings of losing control, being “stuck in a maze” or “having no escape from the illness” have been reported in parents of children with TSC (73). Similarly, parents of children with PMS also had little hope for the future, their hopes were diminished, and they preferred to live from day to day (53).

In relation to TSC, carers show more depressive symptoms than the general population, understanding that neuropsychiatric disorders, behavioral problems, and seizures significantly increased parental stress (65, 72). Parents are anxious about the appearance of new symptoms and side effects of treatments and fearful about the future and the progression of symptom, especially if they are unable to care for their child or if they die (65, 66). These parents feel emotionally overwhelmed, overburdened by the intensity of care for psychiatric and neurological problems, and feel that they have scarce psychological support (72), Similar results were found for PMS, where thinking about the future of their child's care and leaving the responsibility of caring to the siblings caused parents distress (53).

Our results show differences between the sleep disturbance data from the questionnaire and the absence of parental accounts. Hesdorffer et al. (89) showed how caring for and continuously monitoring children with DEE affected sleep quality (aOR 95% CI, 1.7–2.6) and caused fatigue (aOR 95% CI, 1.5–2.2) in carers. Fatigue and poor sleep quality, in turn, increased carers' risk of anxiety (aOR 95% CI, 3.6–6.0) and depression (aOR 95% CI, 2.8–6.0). Gonçalves et al. (90) showed that carers of children with Dravet had a higher incidence of depression and anxiety compared to carers of other patients with epilepsy. These problems were associated with carer fatigue and sleep disturbance.

### 4.4. Strengths and limitations

The presence of multiple genetic variants and the small number of affected individuals were the main challenges of this study, which limited recruitment and consequently may limit the generalization of this study. Another possible limitation is that all participants were recruited from patient associations. This means that participants may have access to more support and information about managing the DEE disorders than families who are not engaged with the patient associations. Although these results should be treated with caution, the strength of this study is that it is the first study to describe the reality of parents of children with STXBP1, TSC and SYNGAP1, using a mixed methods approach with multiple data collection and analysis strategies to increase the depth and credibility of the findings (61, 91). The advantage of a mixed concurrent design is that it enables the integration of data of different natures simultaneously, unlike previous studies carried out with only one type of quantitative (7, 8) and/or qualitative methodology (12). An attempt to cover the objectives of the present study by conducting an observational study and a qualitative study separately, would mean that each method would present its own partial view of the results without integrating the responses to the questionnaires and the individual narrative perspective of the parents. This mixed concurrent design allows for a greater understanding of the phenomenon (having children with DEE) by being able to compare and identify similarities and differences in the responses and behaviors of parents of children with DEE using both qualitative and quantitative data simultaneously (15, 16). The simultaneity of the information is the advantage of using the concurrent mixed design to achieve our objective, since the sequential mixed designs (exploratory or explanatory), must first conduct a phase of the research (QUAN and/or QUAL) and achieve its objectives, to proceed with the next methodological phase, and finally integrate the data (15, 61, 91). In the exploratory approach, the second method (qualitative/quantitative) is needed to answer a research question within a larger quantitative or qualitative study. Moreover, explanatory sequential designs use the results of first method (usually qualitative) to inform the second method (identify variables, develop instrument-QUAN) (15).

In conclusion, our results help to analyze and compare the quality of life, the impact on the family and the self-perceived mental health of parents of children with TSC, STXBP1 and SYNGAP1, with their first-person narrated experiences. This study also identifies areas for improvement, differences among the questionnaires and helps to understand the context in which parents responded to the questionnaires. These results may help professionals to identify gaps in care and improve support and services for these families. These dimensions should be further studied in other genetic variants of DEEs. Future lines of research include: (a) mixed studies focusing on as many genetic variants as possible (e.g., CDKL5, SHANK3, DUP15, SCN1A, KCNQ2, GNAO1, PCDH19) in order to have a quantitative and qualitative comparative basis to integrate and understand the disease from the parents' perspective, (b) including the perspective of professionals who care for children and parents in these studies, and (c) longitudinal studies that consider psychological aspects of caregivers, especially sleep quality, to verify if they are related risk factors for long-term disability of the parent or child, worse family quality of life or worse prognosis of the affected child.

## Data Availability

The raw data supporting the conclusions of this article will be made available by the authors, without undue reservation.
