# Supplementary material for: Quality of life, socioeconomic and psychological concerns in parents of children with tuberous sclerosis complex, STXBP1 and SYNGAP1 encephalopathies: a mixed method study
Source: Front Pediatr. 2023 Nov 9;11:1285377. doi: 10.3389/fped.2023.1285377 (PMC10665567; doi:10.3389/fped.2023.1285377)
Supplement: Supplementary file 1 [file Datasheet1.pdf]

Supplementary Material

Supplementary Figure

Supplementary File 1. S1. Convergent mixed method study process.

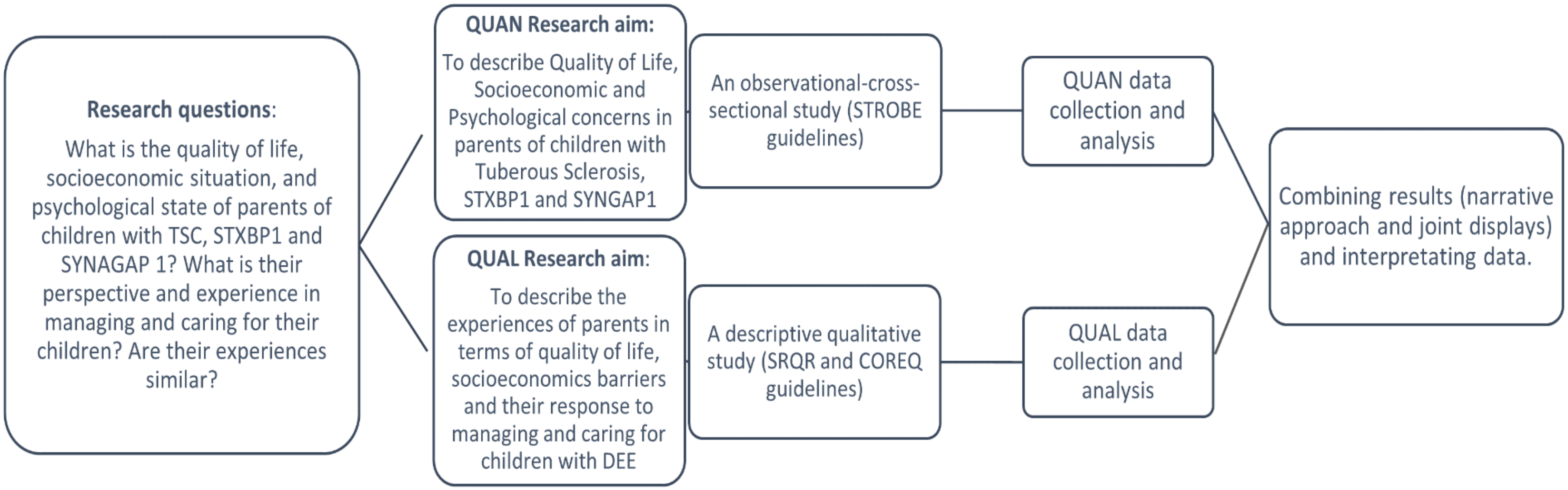

Supplementary Table

Supplementary File 2. S2. Data analysis and coding procedure

**How**	**Instruments & researchers**	**Stage 1: Identify codes**	**Stage 2: Creation of first code book**	**Stage 3: Identify categories**	**Stage 4: Creation of second code book**	**Stage 5: Identify themes**
Use of an excel template, with hyperlinks, shared on OneDrive (Microsoft), and face-to-face and Teams (Microsoft) work meetings.	Interviews	Relevant text excerpts, and identification of codes.	List of codes per participant and per data collection instrument. Unified in the same matrix.	Grouping of codes, identification and definition of categories.	Review and unification of codes and categories. List of codes by participants and by data collection instruments. Unified in the same matrix.	Joint team meetings to combine the results of the analysis. Final themes were displayed, combined, integrated, and identified.
	Researchers	DPC, MSPJ	DPC, MSPJ	DPC, MSPJ	DPC, MSPJ	DPC, MSPJ, ASNG, AAS, LLF

Supplementary Table

Supplementary File 3. S3. Trustworthiness criteria.

Criteria	Techniques used
Credibility	Researcher triangulation: each interview was analysed by two researchers. Team meetings were then held in which the analyses were compared and themes were identified.
	Member checking: this consisted of asking participants to confirm the data collected. All participants were offered the opportunity to review the audio and/or video recordings to confirm their experience. None of the participants made any additional comments.
Transferability	In-depth descriptions of the study were made, detailing the characteristics of the investigators, participants, sampling strategies, and data collection and analysis procedures.
Dependability	Audit by an external researcher: an external researcher evaluated the study's research protocol, focusing on aspects related to the methods applied and the study design. The external auditor was not involved in the health care of the children.
Confirmability	Researcher triangulation, data collection and analysis triangulation.<br>Researcher reflexivity was encouraged through reflective reporting and description of the rationale for the study.

Supplementary Table

Supplementary File 4. S4. Joint display of quotes organised by study dimensions.

Dimensions	Qualitative accounts/quotes
Quality of life	**Family relationships**: *"I had a close relationship with my in-laws. However, when the epilepsy first appeared, the relationship deteriorated. My in-laws are of the mind-set that if you have a sick child you lock them in the house and don't let people see them. An old-fashioned way of thinking that I completely disagree with." (STXBP1, A2)*<br><br>**Rejection**: *"Neither the world nor the people around us are prepared for it (...) My daughter has far fewer opportunities than the other children". (SYNGAP1, C1)*<br><br>**Family support**: *"My immediate family, parents, brothers, sisters, brothers-in-law, sister--in-law and parents-in-law are very supportive. They are available to help with everything, school, doctor's appointments. It wouldn't be the same without them." (STXBP1, A5)*<br><br>**Relationship with siblings:** *"Her older sister has been with her for 5 years and they have a very close relationship (...) She knows how to handle her very well. It gives me peace of mind to see them so close." (TSC, B3)*<br><br>**Importance of associations**: *"The Association is essential, there are people who are going through the same thing as you, they understand and support you. We are all fighting for the same thing." (STXBP1, A1), "The Association clarified may of our doubts and banished many fears. Every time we are about to take a decisive step in the illness, I contact them." (TSC, B1)*

	**Relationship with health care professionals**: "*I felt that they wouldn't listen to me, that they didn't look at or interact with my daughter. I told them that I couldn't trust them. I needed someone to really listen to me." (SYNGAP1, C4)*<br><br>**Obstacles**:<br><br>• Receiving aid: *"The problem is that if we have two salaries and we are employed, we don't qualify for benefits, even if we can't make ends meet." (STXBP1, A2)*<br>• Bureaucracy: *"We applied for disability benefits. They gave it to us a year later. My child's file was lost, they rejected it, we appealed, but we had to start from scratch. I had a hard time with the administrative procedures, the help they gave us." (TSC, B4)*<br>• Waiting lists: *"The worst thing is the waiting list, for some tests you must wait one year. The care is slow and the illness progresses very quickly." (STXBP1, A2)*<br>• Hiring private professionals: *"They give you early intervention services until the age of 5. After that, they are overburdened and they give you an appointment once every two months. For my daughter it wasn't enough and we had to seek external help from psychologists, physiotherapists, and speech therapists." (SYNGAP1, C1)*
Impact on the family	**Everything centred around the child**: *"The family's priority is the child, everything revolves around them, their care, consultations, recovery. It is a dynamic that is difficult to change." (STXBP1, A2)*<br><br>**Constant vigilance**: *"It is intense and requires a lot of dedication. You must monitor the child a lot, it's a constant daily duty." (TSC, B3)*<br><br>**Physical demands**: *"It depends on the size, the weight and if you need transport. If you want to go for a walk, I can't just go anywhere, because, although she has a motorised wheelchair, it's heavy and difficult for one person to handle alone." (STXBP1, A5), "The problem is when you are away from home. If she throws a fit on the side of the road, it's*

	very dangerous, she's getting bigger and bigger and she's getting stronger and stronger. We must go out together because I can't control her on my own." (SYNGAP1, C1)<br><br>**Behavioural management problems**: *"What worries us the most is her behaviour, it's the most difficult aspect to manage and it's what affects us the most emotionally. She has a low tolerance to frustration and her reaction is strong and violent. She bites us, pulls our hair, hits us sometimes. It's difficult to manage emotionally, because your own daughter is attacking you." (SYNGAP1, C1)*<br><br>**Change in social relations**: *"We have stopped going out for dinner, or going out for a beer, because it coincides with the time that we have to administer her midday medication. I have stopped meeting people who feel uncomfortable with my daughter or don't accept her." (TSC, B4)*<br><br>**Impact on siblings:** *"I can see that it is a big burden for her, being her big sister. She is still a child who has her needs too. She also suffers from the situation." (TSC, B3)*<br><br>**Difficulty finding carers**: *"You make plans, but I can't find anyone to stay with my son. They're afraid to stay with him, he's a big boy and they know he could have a breakdown and they don't dare stay alone with him. That's the way it is, I must accept it." (STXBP, A2)*<br><br>**Learning with your children:** *"It has changed my view of people and the world. I have become more respectful and flexible with situations and people". (SYNGAP1, C1)*<br><br>**Impact on the couple:** *"It's complicated because it takes up a lot of your time, there are a lot of fights, but in reality, it has brought us even closer together. It has made us stronger." (STXBP1, A3)*

	**Having another child:** *"The decision not to have any more children has not been made, but I wouldn't do it again. Because you always have the doubt of whether there is a problem, or whether I will have time for everything. Most of all, being able to do everything". (STXBP1, A3)*<br><br>**Financial impact:** "*It's often a matter of making a living to pay for all the treatments, and if you can't, then that's it. We have always had to choose because we didn't have money for everything. We even had to take out a loan to cover all the costs of care*". (*TSC, B4)*<br><br>**Reduced working hours:** "*We had no choice, we had to make time to care for her. I had to ask for a reduction in my working hours. On the one hand, I needed time, but on the other hand we were struggling financially. It was frustrating*." *(SYNGAP1, C4)*
Psychological factors	**Responsibility:** "*You always wonder if you did something to cause this. To what extent am I responsible for the illness...". (SYNGAP1, C4)*<br><br>**Grieving:** "*The family has accepted it and they have been accepting it to a greater or lesser extent. Each person has their own way of coping with it psychologically, each person grieves in their own way*". *(SYNGAP1, C1)*<br><br>**Hopes for the future:** "*I have no hopes because I know they don't exist. Do I hope I will have a normal life? Yes. Hopes that he will get better or be cured? No. I don't think so*". *(TSC, B4)*<br><br>**Hard but rewarding experience:** "*It is an intense and difficult experience, but also rewarding, because they are children who give you all their love, without any ulterior motives, they have no evil intentions. They need us and convey this to us, and that makes me very happy*". *(SYNGAP1, C1)*

	**Concern:** "*I'm always worried, it's a feeling of fear and anxiety, that something might happen to the child and we'll have to go to hospital. Basically, I can't relax.” (STXBP1, A2)*<br><br>**Guilt:** " *Her brother's studies are going well, and sometimes I feel guilty for not being with him enough and focusing on his sister who is sick*”. *(TSC, B2)*<br><br>**The future of your sick child:** “*Putting my son in an institution scares me a lot. Right now, I feel sick at the thought of it. We will leave that responsibility to the future, when we are too old or he becomes unmanageable. We don't even want to think about it right now*". *(SYNGAP1, C3)*<br><br>**Overwhelmed:** "*It's a lot of stress, because I have the role of mother, the role of the head of the household and the role of a business owner. You must take care of everything, there are days when I can't cope*". *(TSC, B4)*<br><br>**Uncertainty:** "*As it is a very unknown illness, they don't know anything, and you don't know how far you can go, or what to expect, you don't know anything. All this uncertainty is very hard.*” *(STXBP1, A5)*<br><br>**Loneliness:** "*I often feel alone. Because your family is far away, because you can't ask them for help, and you have to experience the illness to understand it*". *(STXBP1, A1)*<br><br>**Emotional crisis:** "*You have to experience it, it's easy to judge. When he was angry, he made our life unbearable, because if he was uncomfortable, he made the world uncomfortable. It felt like if he was uncomfortable he would constantly shout at us and kick us. Sometimes I couldn't help hate and resent my own son.*" *(SYNGAP1, C3)*

	**Feelings about reduced working hours:** "*It makes me angry; I have studied a lot to stay at home. You lose the chance to evolve at work, to grow socially. You lose many things, it's hard to take the step and get used to it, you feel useless, and it affects your self-esteem*". *(STXBP1, A5*)

Supplementary Table

Supplementary File 5. S5. Medians (interquartile range) of the questionnaires related to parent´s quality of life and impact.

			Total sample (n=20)	STXBP1 (n=10)	SYNGAP1 (n=5)	Tuberous Sclerosis Complex (n=5)
Short-Form Health Survey (SF-12-v2)		Mental component	51.0 (8.6)	51.6 (8.1)	48.7 (12.1)	46.4 (5.4)
		Physical component	54.1 (12.9)	55.2 (14.6)	53.2 (4.6)	44.8 (13.8)
Beach Center Family Quality of Life (BCFQOL) Scale	Importance	Family Interaction	4.8 (0.4)	4.8 (0.3)	4.83 (1.2)	4.83 (0.2)
		Parenting	4.7 (0.8)	4.7 (0.7)	4.67 (0.8)	4.7 (0.5)
		Emotional Well-being	4.8 (1.3)	4.8 (1.3)	4.75 (1.3)	5.0 (0.5)
		Physical / Material Well-being	4.8 (1.0)	4.9 (0.8)	4.8 (1.0)	4.60 (1.0)
		Disability-Related Support	4.9 (0.8)	5.0 (0.3)	5.0 (0.3)	4.3 (1.0)
	Satisfaction	Family Interaction	3.9 (0.8)	3.9 (0.8)	3.8 (1.3)	4.0 (0.5)
		Parenting	3.8 (1.5)	3.7 (1.2)	4.0 (1.8)	4.0 (0.7)

		Emotional Well-being	2.9 (0.8)	3.3 (0.8)	2.8 (0.5)	2.8 (0.5)
		Physical / Material Well-being	3.8 (0.6)	4.1 (0.6)	3.6 (0.8)	3.8 (0.4)
		Disability-Related Support	3.6 (1.3)	3.6 (1.0)	4.5 (0.7)	3.3 (0.3)
Impact on Family Scale		Total impact (96 points)	44.0 (18.0)	44.0 (7.0)	44.0 (3.0)	37.0 (24.0)
		Family-social Impact (36 points)	20.5 (9.0)	19.5 (10.0)	21.0 (1.0)	15.0 (9.0)
		Personal impact (24 points)	11.0 (5.0)	12.5 (9.0)	11.0 (1.0)	10.0 (6.0)
		Experience with the illness (20 `points)	8.0 (2.0)	8.0 (3.0)	8.0 (1.0)	8.0 (1.0)
		Economic burden (16 points)	6.5 (4.5)	6.5 (5.0)	4.0 (4.0)	7.0 (3.0)
Impact of Paediatric Epilepsy Scale (IPES)			13.0 (22.0)	13.0 (19.0)	10.0 (22.0)	22.5 (7.0)
